# Effect of Nitrogen-Doped Graphene Oxide on the Aging Behavior of Nitrile–Butadiene Rubber

**DOI:** 10.3390/polym11101637

**Published:** 2019-10-10

**Authors:** Songbo Chen, Tianxiang Li, Songhan Wan, Xing Huang, Shuwei Cai, Xianru He, Rui Zhang

**Affiliations:** 1 School of Materials Science and Engineering, Southwest Petroleum University, No. 8 Xindu Avenue, Xindu District, Chengdu 610500, China; 2 Institut für Physik, Universität Rostock, Albert-Einstein-Str. 23-24, 18051 Rostock, Germany; rui.zhang@uni-rosotck.de

**Keywords:** nitrile-butadiene rubber, nitrogen-doped, aging resistance, graphene oxide

## Abstract

Nitrogen-doped graphene oxide (GO), namely, NG, was prepared by o-phenylenediamine (OPD) grafting onto GO. The structure and morphology of NG were characterized by FITR, XRD, SEM, EDS, Raman spectroscopy, and TGA. OPD was linked to the GO surface by covalent bonds, and the absorption peak of the C=N bond in the phenazine structure was identified in the FITR spectra. The aging resistance properties of nitrile-butadiene rubber (NBR)-NG composites was investigated by mechanical testing, before and after aging. The resistance of the NBR/NG composites with the addition of 3 phr NG fillers was the highest. The aging mechanism was investigated by TGA-DSC, DMA, equilibrium swelling testing, and ATR-FTIR. The results showed that NG could effectively inhibit chain cross-linking in NBR.

## 1. Introduction

Nitrile-butadiene rubbers (NBR) has been widely applied in the fields of automotive, coatings, petroleum and printing, because of its excellent oil resistance, wear resistance and elastic properties [1,2,3]. However, due to the presence of abundant C=C bonds in rubber, NBR suffers from aging in air, acid and alkali media, which limits its service life. To improve aging resistance properties, physical blending and chemical modification are deemed essential. Generally, physical blending is more popular because of its easy operation and low environmental pollution [4,5,6,7,8].

Graphene has received much attention as polymer filler, because of its gas barrier property, excellent physical and chemical property and high conductivity property [9,10]. However, it establishes strong inter-layer interactions in polymer matrices, which cause its agglomeration. Graphene oxide (GO), a derivative of graphene, presents many oxygen-containing functional groups, which enable to weaken the inter-layer interactions of graphene and provide a great number of active sites. However, the thermal stability of GO is poor. As a consequence of the removal of oxygen functional groups during vulcanization, GO produces free radicals and gases, which affect the comprehensive properties of rubbers. Thermal reduction of GO can avoid this situation but will increase GO dispersion in the rubber and affect the mechanical properties and aging resistance of composites.

Aromatic amine antioxidants are often used as polymer anti-aging agents [11]. They can terminate the free-radical chain reaction in the aging process of rubber [12]. However, most commercial antioxidants are small molecular substances, which can easily migrate from the bulk to the surface of a rubber during vulcanization and use, leading to environmental pollution and decrease of the aging resistance. Grafting these substances onto GO can avoid their migration and increase the dispersion of GO in matrices.

In our previous work, by grafting p-phenylenediamine (PPD) onto GO, the aging resistance properties of NBR/GO composites were effectively improved [8]. However, the amino group of PPD is in a para-position on the benzene ring and has a lower steric hindrance effect. This makes PPD crosslinking between GO layers easy, which, to some extent, affects the dispersion of GO. The molecule o-phenylenediamine (OPD) has greater steric hindrance, because its amino groups are adjacent to each other. Herein, OPD was selected as a grafting modifier of GO. Because of the greater steric hindrance effect, the crosslinking between GO layers could be avoided, and the dispersion of GO improved as a result of in situ reduction by OPD. At the same time, a phenazine structure with better anti-aging properties was obtained during the grafting process. The better anti-aging structure combined with the better dispersion, increased the anti-aging efficiency. It is important to implement green anti-aging techniques of NBR/NG composites, conferring excellent aging-resistance properties.

In this paper, nitrogen-doped graphene oxide (NG) was prepared by modifying graphene oxide with OPD through a hydrothermal method. The structure and morphology of NG fillers were characterized by FTIR, XRD, TGA, SEM, and EDS. NBR/NG composites were prepared by mechanical blending. The aging-resistance properties of NBR composites were tested. Moreover, the aging mechanism were investigated by TGA-DSC, DMA, ATR-FTIR and equilibrium swelling testing.

## 2. Experimental

### 2.1. Materials

Graphite oxide (industrial grade) was purchased from the sixth element Mstar Technology Ltd. in Changzhou, China. OPD, zinc oxide (ZnO), stearic acid, N-Phenyl-2-naphthylamine (antioxidant D), sulfur, and dibenzothiazole disulfide (DM) were purchased from Chengdu Kelong Chemical Reagent Factory, Chengdu, China. NBR (2907, acrylonitrile content 29%) was purchased from PetroChina Lanzhou Petrochemical Company, Lanzhou, China.

### 2.2. Preparation of Nitrogen-Doped Graphene Fillers

NG fillers were prepared by a hydrothermal method. Briefly, 1g of GO dispersions in 200 ml deionized water, named A, and 1 g of OPD dispersions in 50 ml ethanol, named B, were mixed by ultrasound for 30 minutes, and then the reaction was carried out in a hydrothermal reactor at 180 °C for 12 h. The product was vacuum-filtered, washed with ethanol and deionized water for several times, and then freeze-dried to obtain NG fillers.

RGO (reduction graphene oxide) fillers were prepared as the contrast samples under the same hydrothermal reaction conditions. The preparation was as follows. A deionized-water dispersion of 1 g of GO was mixed by ultrasound for 30 minutes, and then the reaction was carried out in a hydrothermal reactor at 180 °C for 12 h. The product was vacuum-filtered, washed with ethanol and deionized water for several times, and then freeze-dried to obtain RGO fillers.

### 2.3. Preparation of NBR/NG Composites

The basic formulation of the NBR composites is shown in Table 1. The above-mentioned drugs were mixed uniformly on a twin-roll open mill. Then, the compounds were cured for 30 minutes by a flat vulcanizing machine at 150 °C and 10 MPa.

### 2.4. Characterization

The functional groups of NG fillers were analyzed by Fourier Transform Infrared Spectrometer (Nicolet IS50, Thermo Fisher Scientific, Waltham, MA, USA). The spectra were recorded by scanning the pellets in the range from 500 to 4000 cm^−1^ with a resolution of 4 cm^−1^.

NG fillers were analyzed by X-ray diffraction (X’Pert PRO, PANalytical B.V., Almelo, The Netherlands), under the test conditions of Cu Ka radiation (λ = 1.54 Å), at a step length of 4°/cm and a scanning angle of 5–70°. 

Raman spectroscopy was carried out by a DXR (Thermo Fisher Scientific, Waltham, MA, USA) with a laser wavelength of 532 nm.

Scanning electron microscopy (ZEISS EV0 MA15, Carl Zeiss optics co., LTD, Oberkochen, Germany) was used to observe the micro-morphology of the composite fillers, the cross section morphology of the composite rubbers, and the dispersion of the composite fillers in the polymer matrix. 

The viscoelastic properties of the composite rubber were characterized by dynamic mechanical analyzer (Q800, Waters China Ltd., Shanghai, China) in double-cantilever beam mode at a heating rate of 3 °C/min, an amplitude of 15 µm, and a frequency of 1 Hz. Moreover, the length, width, and thickness of the sample were 25, 6, and 2 mm, respectively. 

The thermogravimetric curves of the NG fillers were determined by thermal analyzer (TGA/SDTA851E, Mettle Toledo, Zurich, Switzerland) in a nitrogen atmosphere at a heating rate of 10 °C/min in a temperature range of 50–800 °C. 

A synchronous thermal analyzer (STA449F3, NETZSCH, Bavaria, Germany) was used to characterize the aging mechanism of rubber in an oxygen atmosphere at a heating rate of 10 °/min in a temperature range of 40–800 °C.

The crosslink density of the NBR composites was determined using an equilibrium swelling method. An NBR (m_1_ = 1 g) sample was immersed in 100 ml toluene for 72 h until reaching swelling equilibrium. Toluene on the surface of the sample was eliminated, and the sample’s mass was recorded as m_2_. The mass of the sample was then recorded as m_3_ after drying at 80 °C for 36 h. The terms in Equation (1) and Equation (2) are defined in Table 2.

(1) $$V_{r}=\;-\frac{1}{V}\left[\frac{{\mathrm{Ln}(1-V}_{2\;}){+V}_{2}{+V}_{3}{+\chi v}_{2}^{2}}{V_{2}^{1/3}{-V}_{2}/2}\right]$$

(2) $$V_{2}=\frac{m_{3}/\rho }{m_{3}/{\rho +(m}_{1}{+m}_{2})/\rho_{s}}$$

## 3. Results

### 3.1. Structure and Morphology of NG

**Figure 1:**
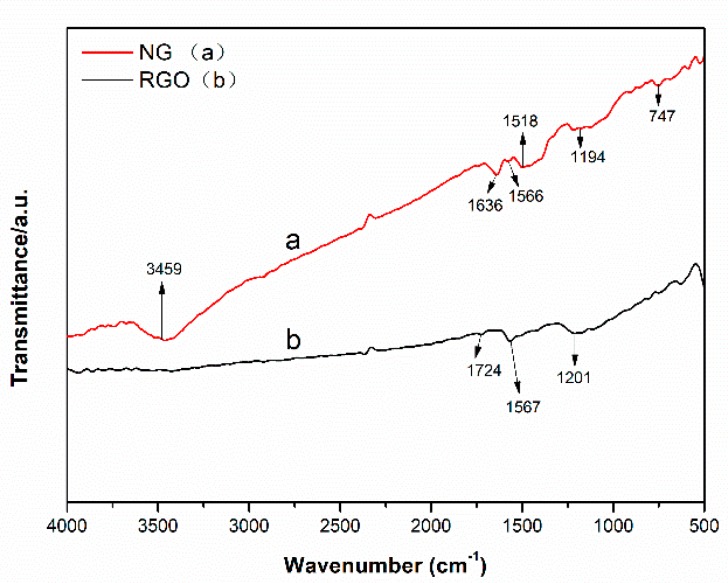
FTIR spectra of NG and RGO fillers.

**Figure 2:**
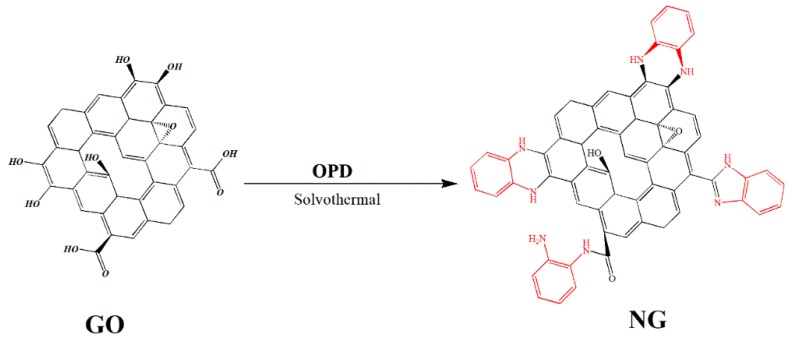
Diagram of the nitrogen-doping reaction process. GO: graphene oxide, OPD: o-phenylenediamine.

The FTIR spectra of the NG and RGO fillers are shown in Figure 1. The peaks at 1724 cm^−1^ and 1201 cm^−1^ in the RGO curve (Figure 1b) correspond to the stretching vibration absorption peak of the carbonyl group and epoxy group, respectively [13]. Both peaks disappear in the NG curve (Figure 1a). Because of the reaction between oxygen groups on GO and –NH_2_ on OPD [14], the absorption peaks of the phenazine structure were recorded at 747, 1194, and 1566 cm^−1^ [15]. According to the FTIR results, the OPD grafting process and the structure of NG were determined, as shown in Figure 2. 

**Figure 3:**
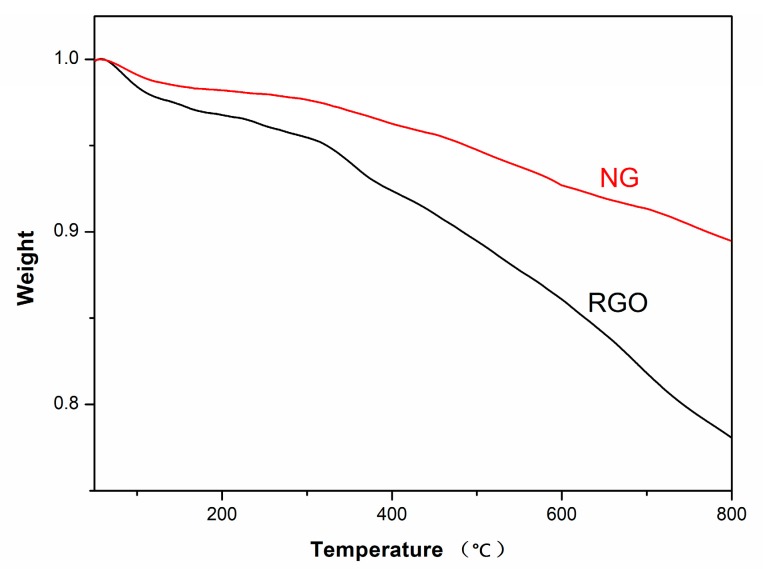
TGA curves of the RGO and NG fillers.

The TGA curves of RGO and NG are shown in Figure 3. The weight loss of RGO at 800 °C was 22%, caused by the decomposition of residual oxygen groups. The weight loss of NG was 11%, smaller than that of RGO. The results showed that the thermal stability of NG was better than that of RGO, because the thermal stability of nitrogen-containing functional groups and of the phenazine structure of NG is higher than that of the oxygen-containing functional groups of RGO [2,14].

**Figure 4:**
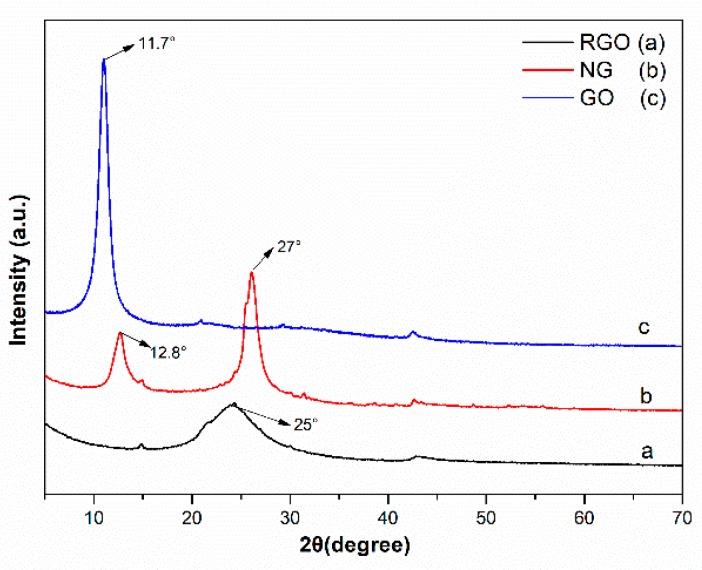
XRD pattern of GO, NG, and RGO fillers.

The XRD patterns of RGO, GO, and NG are shown in Figure 4. Compared to the characteristic spectrum of GO, the peak shift observed in the RGO spectrum indicated that hydrothermal treatment caused the reduction of GO. In the spectrum of NG, the peak at 12.8° corresponds to the structures of phenazine and quinone in NG. The peak at 27° is sharper than the corresponding one of RGO, due to the more regular stacking structure of graphene in NG. Moreover, the peak shifted to a large angle, because of the reduction of GO under the combined action of hydrothermal conditions and OPD.

**Figure 5:**
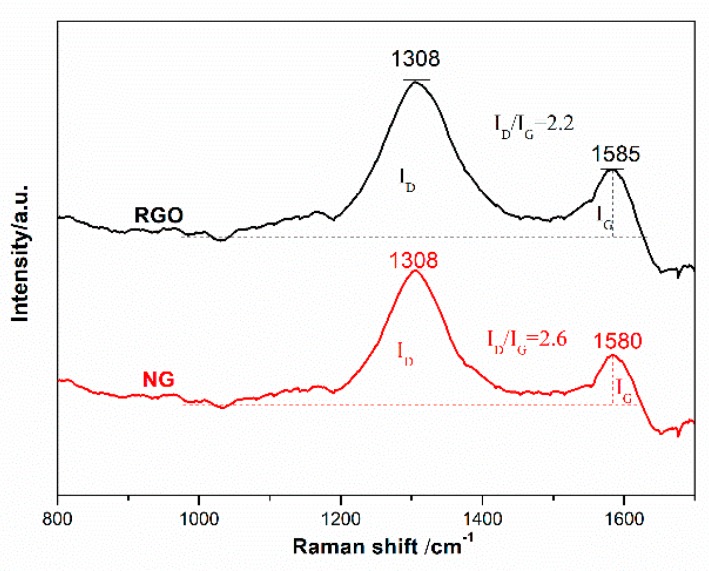
Raman spectra of NG and RGO.

The Raman curves of NG and RGO are shown in Figure 5. The position and intensity of these peaks provide information on the type of defects in the graphitic material. The D peak corresponds the structural imperfections created by the attachment of hydroxyl and epoxide groups. The G peak represents the in-plane stretching vibration of sp^2^ hybridization [16,17]. The I_D_/I_G_ of NG was 2.6, higher than that of RGO. It indicated that the phenazine structure, formed by OPD and GO, destroyed the surface regularity of GO and increased its defects [18,19].

**Figure 6:**
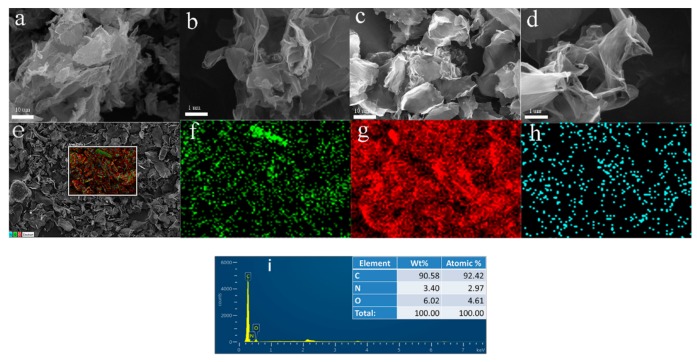
SEM images of RGO at 5000× (**a**) and 50,000× magnification (**b**); SEM images of NG at 5000× magnification (**c**) and 50,000× magnification (**d**); (**e**) EDS images of NG; distribution diagram of the elements O (**g**), C (**h**) and N; (**i**) diagram of the content of NG elements.

The SEM and EDS images of NG and RGO are shown in Figure 6. RGO (Figure 6a) showed irregular stacking and agglomeration. The layers of NG (Figure 6b) were more convoluted and looser, and the stacking structure was more regular, which was consistent with the conclusion of the XRD analysis. In the partial enlargements shown in Figure 6c,d, the sheet of NG appeared more curly and thinner, because OPD present in the solvothermal reaction could effectively prevent the self-stacking behavior of NG [20]. The distribution patterns of nitrogen, oxygen, and carbon are shown in Figure 6f–h, respectively. Nitrogen, oxygen, and carbon were uniformly distributed on the surface of NG, and the nitrogen content on the surface reached 3.4%.

### 3.2. Properties of NBR Composites

**Figure 7:**
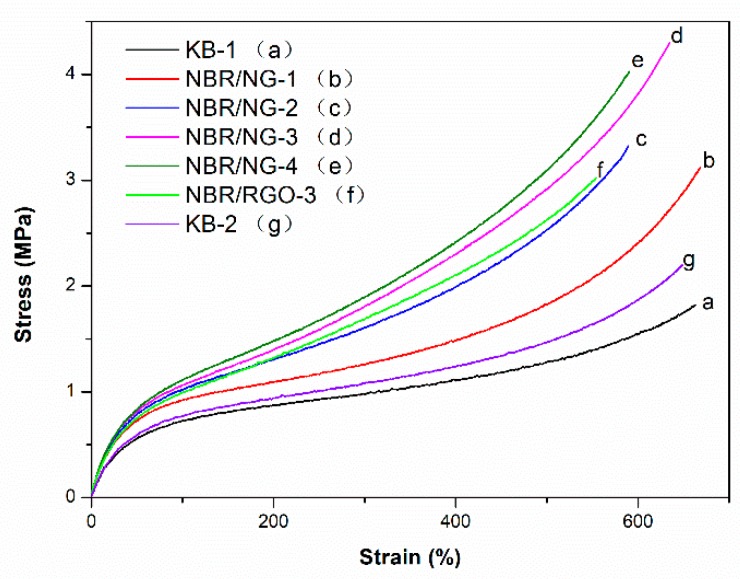
Stress-strain curves of NBR composites.

The mechanical test results of NBR composites are shown in Figure 7 and Table 3. The data in Table 3 are the statistical average values of five parallel samples. The stress–strain curve in Figure 7 is the corresponding curve of the sample approaching the statistical average value. With the addition of NG, the stress at break and elongation at break of NBR/NG composites were higher than those of KB-1 and KB-2. The maximum stress at break of NBR/NG composites was 130.8% higher than that of KB-1. It is worth noting that the slope of the NBR/NG composites’ curves increased sharply after the strain exceeded 350%. On the one hand, this was because the rubber chain had the maximum orientation at the smallest strain as a result of the physical adsorption of NG on the NBR matrix. On the other hand, it was caused by the lamellar structure of NG inhibiting the movement of NBR molecular chains, which hindered the stretching orientation of NBR molecular chains. In addition, the stress at break and the elongation at break of the NBR/NG-3 composite was higher than those of NBR/RGO-3, especially the stress at break, which increased about 40%. This indicated that NG easily formed a percolation network structure in the NBR matrix with less GO, compared to NBR/RGO-3, because the organic modification of GO by OPD could improve its dispersion in NBR, thus enhancing the mechanical properties of NBR composites. 

**Figure 8:**
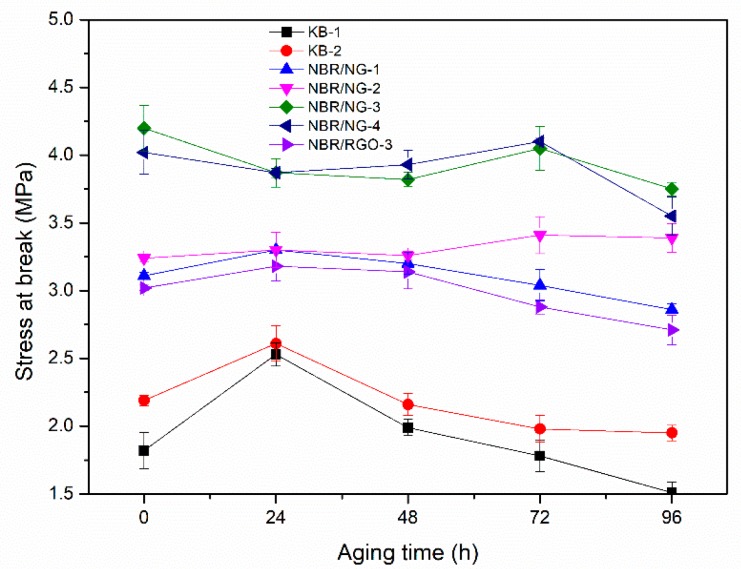
Stress at break of NBR composites at different aging times.

**Figure 9:**
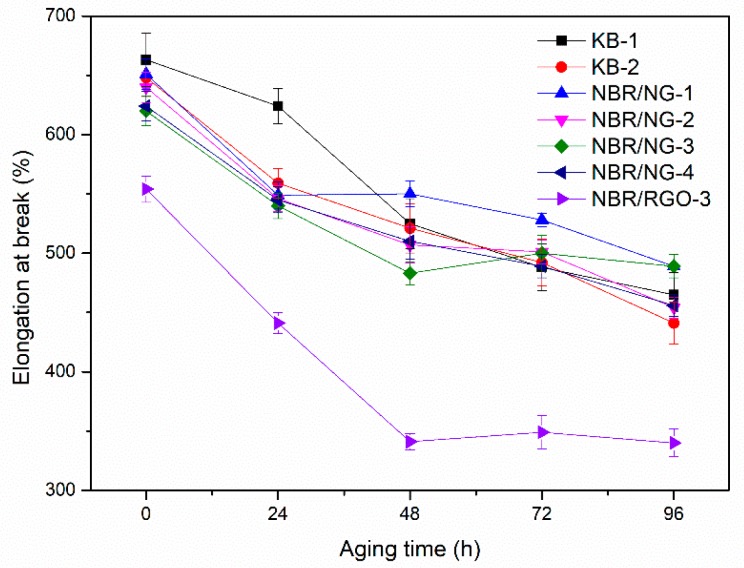
Elongation at break of NBR composites at different aging times.

The test results of stress at break and elongation at break of the NBR composites after different thermal oxygen aging time are shown in Figure 8 and Figure 9. With the increase of the aging time, the stress at break of KB-1 and KB-2 first increased and then decreased. It is possible that the sample continued to be vulcanized and cross-linked in the aging process. However, the stress at break of the NBR/NG composites did not show large fluctuations during the aging process. The elongation at break of all samples showed a downward trend, but the downward trend of the NBR/NG composites was less sharp than that of KB-1 and KB-2. The stress at break and the elongation at break of NBR/NG-3 was obviously higher than that of NBR/RGO-3. This indicated that NG could effectively inhibit the thermal oxygen aging of NBR and enable it to maintain better mechanical properties after thermal oxygen aging. In addition, from our previous work [8], GO contributes less to the improvement of aging resistance of NBR, because it agglomerates easily and cannot form an effective network structure. Besides, it does not have anti-aging groups or the ability to capture free radicals in the NBR chains during the aging process.

**Figure 10:**
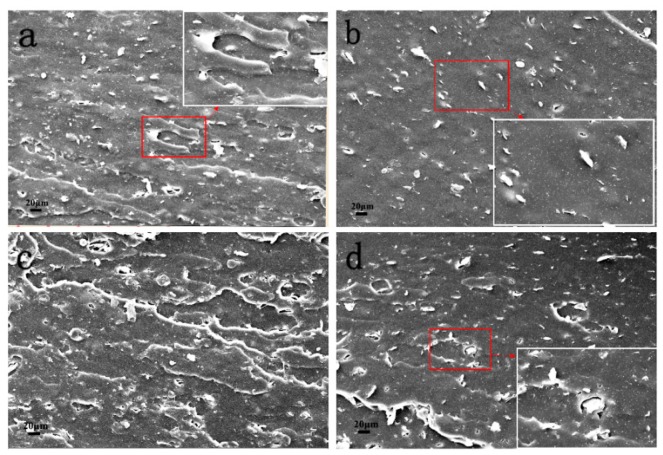
SEM images of the fracture surface of NBR/NG-3 (**a**) before aging and (**c**) after aging; fracture surface SEM images of NBR/RGO-3 (**b**) before aging and (**d**) after aging.

The fracture surface SEM images of NBR/NG-3 and NBR/RGO-3 before and after aging are shown in Figure 10. The fracture surface of NBR/NG-3 before aging (Figure 10a) was rough and some gullies appeared. Because of the strong interaction between NG and NBR matrix. The fracture surface of NBR/RGO-3 before aging was smooth (Figure 10b), and some voids appeared. Because the interaction between RGO and NBR was weak, RGO detached from the NBR matrix during stretching. The fracture surface of NBR/NG-3 after aging (Figure 10c) still presented some gullies. It indicated that the NG and NBR substrates maintained good interactions during the aging process, and no obvious phase separation occurred. It is important to maintain good mechanical properties of NBR/NG-3. Moreover, the NG sheet could block the entry of oxygen during the aging process. The fracture surface of NBR/RGO-3 after aging (Figure 10d) showed obvious pores, which were caused by the phase separation of RGO and NBR matrix during the aging process. These holes reduced the mechanical properties of NBR and also provided channels for the entry of oxygen, accelerating the degradation of the rubber chain.

### 3.3. Reasons for Improving NBR Aging Resistance by an NG filler

**Figure 11:**
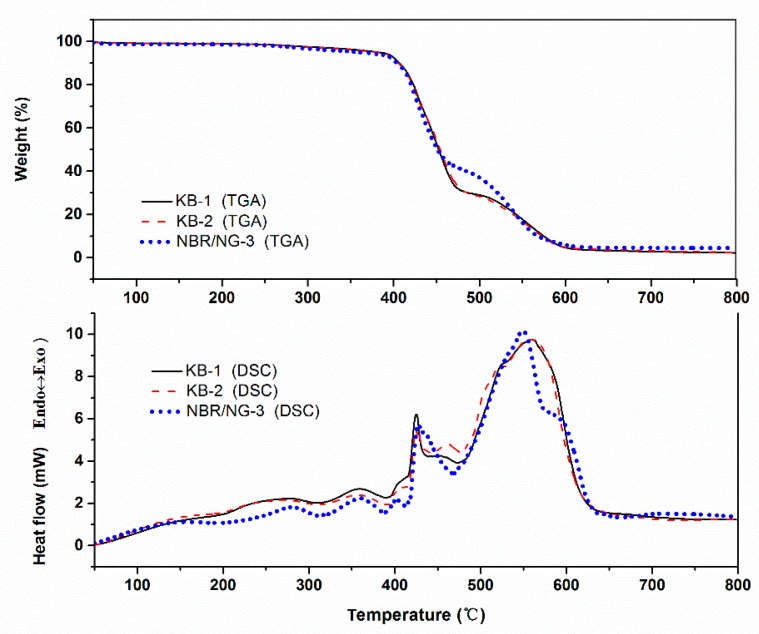
The TGA-DSC curves of KB-1, KB-2, and NBR/NG-3.

The TGA-DSC test results of KB-1, KB-2, and NBR/NG-3 are shown in Figure 11. The thermal weight loss process of NBR shown by the TGA curve occurred mainly in three stages. The first stage (before 400 °C) was mainly the volatilization of small-molecule additives in the rubber and the primary degradation of the rubber chains. The thermal oxygen degradation of the rubber chains occurred in the second stage (400–500 °C) with the greatest weight loss. The third stage (500–600 °C) was the further degradation of the residual molecular chains. The DSC curve also presented three phases. A series of exothermic peaks appeared in the first stage (before 420 °C) because of the oxidation reaction of the molecular chain of NBR. The second stage (420–500 °C) showed an endothermic peak, and the sample presented the highest weight loss rate due to the decomposition reaction of the rubber chains. A distinct exothermic peak appeared in the third stage (after 500 °C), because of the further oxidative degradation of the rubber. A comparative analysis of DSC curves revealed that the intensity of the exothermic peaks in the first stage varied greatly, while the difference in peak intensities in the second and third stages was not obvious. It indicated that the first stage was related to antioxidants and fillers. The KB-1 sample without anti-aging agent and filler presented the most obvious exothermic peak, while the exothermic peak of the NBR/NG-3 sample was the weakest. NG fillers can inhibit the initial oxidation process of rubber, and their anti-aging effect is superior to that of commercial antioxidants, because of the phenazine structure in NG and the gas barrier of GO. However, the overall structure of NBR was destroyed in the middle and late stages of degradation. This indicated that NG had a good inhibitory effect on the initial stage of NBR aging.

**Figure 12:**
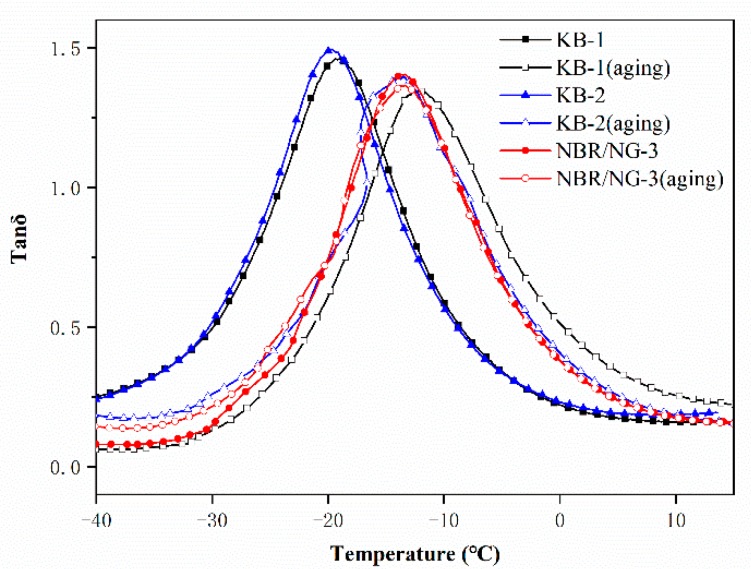
Loss factor (tanδ) curves of KB-1, KB-2, and NBR/NG-3.

The DMA results of KB-1, KB-2, and NBR/NG-3 are shown in Figure 12. The loss factor (tanδ) peak position of NBR/NG-3 shifted to a higher temperature before aging compared to those of KB-1 and KB-2. This indicated that the glass transition of NG was higher than those of KB-1 and KB-2, because NG formed many physical cross-linking points in the rubber and increased the interaction between the NG and the rubber chains [21]. Comparing the changes of the tanδ peak before and after aging, the tanδ peak positions of KB-1 and KB-2 moved to higher temperatures. This indicated that the NBR chain was further cross-linked during aging process, which restricted the movement of the segments and increased the glass-transition temperature of the NBR composites. Moreover, the tanδ peak position of the NBR/NG-3 composite remained almost unchanged. This showed that NG can effectively inhibit further crosslinking of rubber molecular chains, and its anti-aging effect is superior to that of commercial antioxidants.

**Figure 13:**
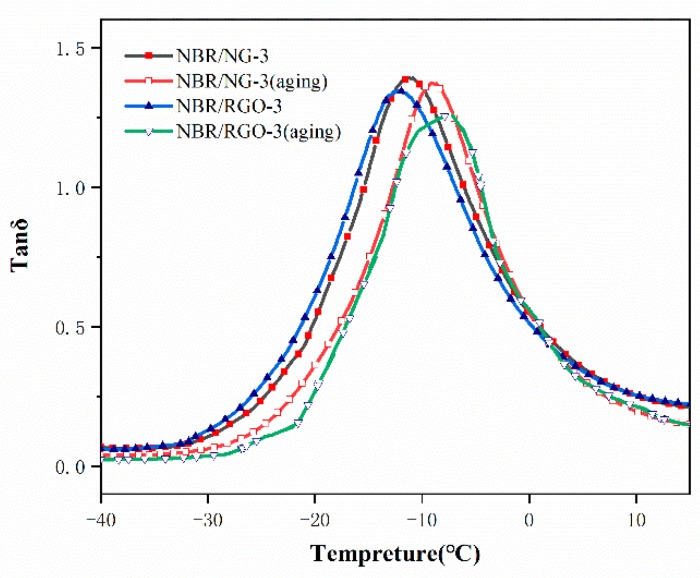
Loss factor (tanδ) curves of NBR/RGO-3 and NBR/NG-3.

The aging phenomenon of NBR/NG composites was not obvious under the aging conditions of 90 °C and 96 h, as shown in Figure 12. Therefore, we used more stringent experimental conditions, i.e., 120 °C for 24 h. The tanδ curves of NBR/RGO-3 and NBR/NG-3 are shown in Figure 13. The tanδ peak position of NBR/RGO is significantly lower than that of NBR/NG before aging, indicating that the glass-transition temperature of NBR/NG was higher, because the dispersion of NG in the rubber was better, and the interactions between NG and the rubber chains increased after organic modification, with the formation of more physical cross-linking points. The tanδ peak position of NBR/NG moved to a higher temperature after aging, because aging in NBR/NG was enhanced. However, the tanδ peak of NBR/RGO migrated to a higher temperature compared to that of NBR/NG. This indicated that the aging phenomenon of NBR/RGO was more obvious, because RGO had no chemical anti-aging functional groups. While the phenolic zine structure of NG, formed by OPD grafted on GO, can capture free radicals in the aging process of rubber, the better dispersion of NG fillers in the matrix can form an effective lamellar barrier. Chemical anti-aging combined with lamellar barrier can significantly inhibit aging of NBR. Therefore, NBR/NG had a better aging resistance than NBR/RGO, which is consistent with the changes in its mechanical properties before and after aging.

**Figure 14:**
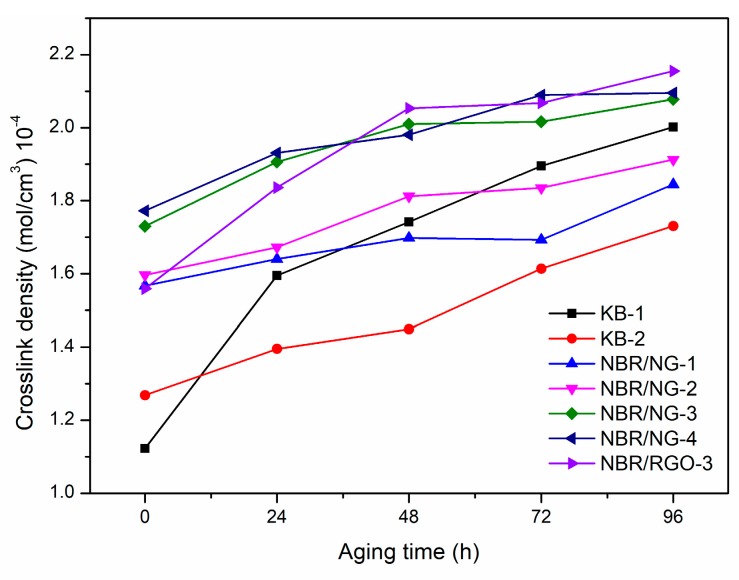
Cross-link density curves of NBR composites.

Essentially, rubber aging is the result of chain scission and cross-linking of molecular chains. The aging of NBR was mainly based on cross-linking reactions, and its cross-link density reflects its cross-link degree, which can be used to explain the changes in the mechanical properties of the rubber during aging. Therefore, the determination of the cross-link density changes in NBR at different aging times could indirectly reflect the aging degree of the rubber, thereby indicating the effect of the filler on the aging resistance of NBR. The cross-link density curves of NBR composites are shown in Figure 14. The cross-link density of each sample gradually increased with the aging time. The increase trend of KB-1 without an anti-aging agent was the most obvious, especially between 0 and 24 h. Significant cross-linking occurred in the early stage of aging. The cross-link degree of KB-2 was significantly lower than that of KB-1, but its cross-link density increased significantly after 48 h, because the volatilization loss of commercial antioxidants led to the decline of the anti-aging effect with aging time prolongation. The cross-link density of NBR/NG composites increased gently because the aniline groups on NG enabled to capture free radicals during the aging process. The strong interaction between NG and NBR made the aniline group less volatile. Moreover, the NG layer could effectively block the entrance and diffusion of oxygen in the matrix. Chemical and physical anti-aging occurred simultaneously, and NG could maintain its anti-aging function for a long time. The cross-link density increasing trend of NBR/RGO-3 was gradually reduced during the aging process, because RGO had no anti-aging function, and its poor dispersion in the matrix led to a poor gas barrier effect.

**Figure 15:**
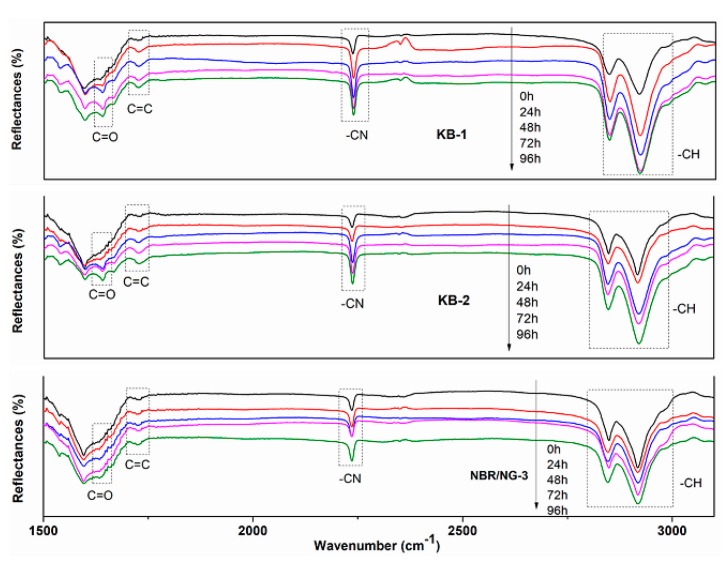
ATR-FTIR spectra of KB-1, KB-2, and NBR/NG-3 at different aging times.

**Figure 16:**
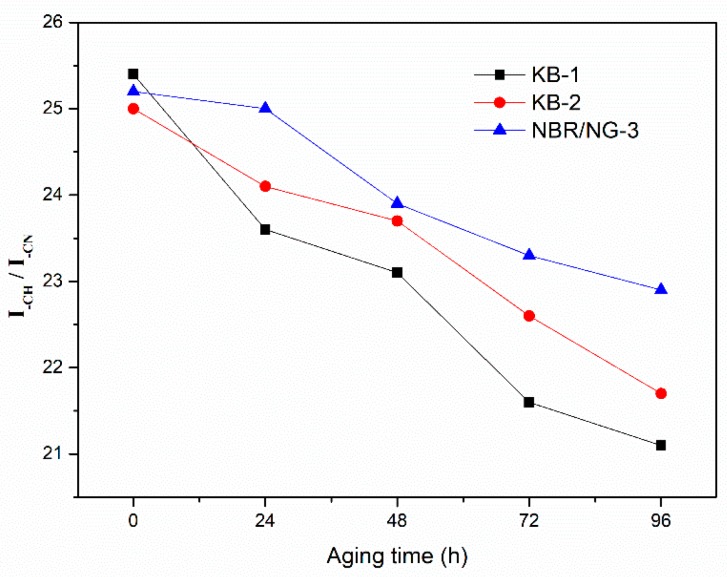
Relative content changes of –CH in KB-1, KB-2, and NBR/NG-3 at different aging times.

**Figure 17:**
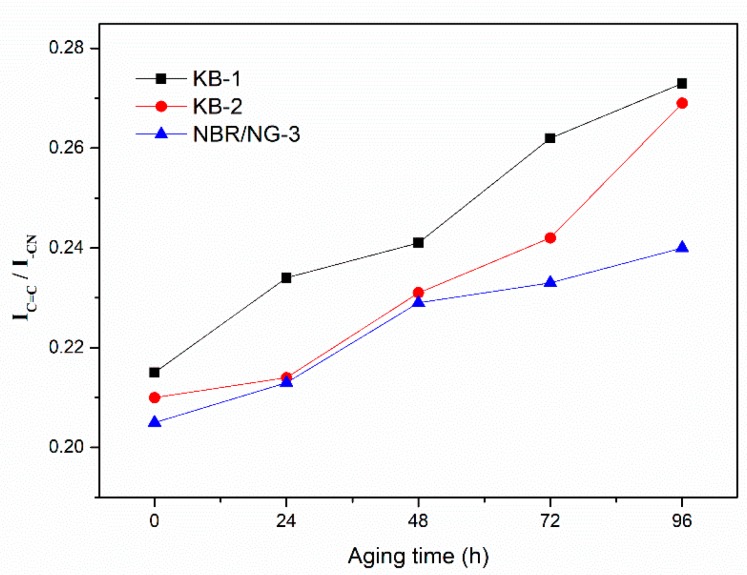
Relative content changes of C=C in KB-1, KB-2, and NBR/NG-3 at different aging times.

**Figure 18:**
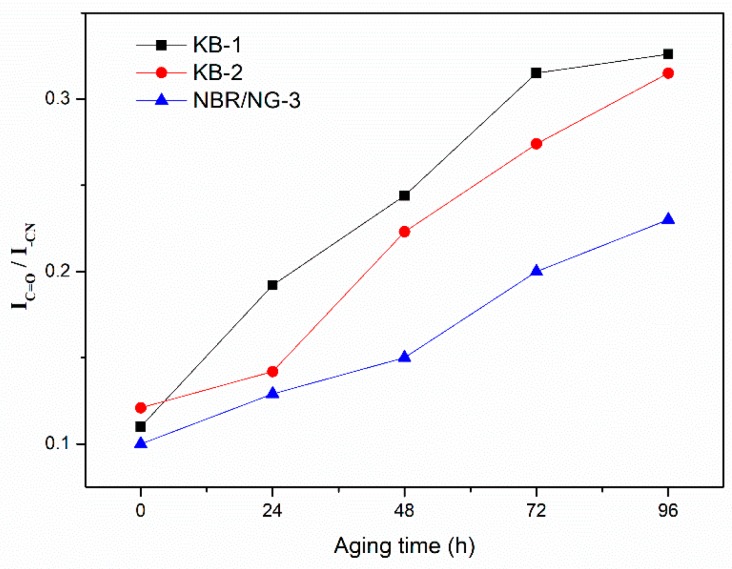
Relative content changes of C=O in KB-1, KB-2, and NBR/NG-3 at different aging times.

DMA and crosslink density tests aimed at determining the change of the degree of molecular chain cross-linking during the aging process. However, the oxidation reaction and chain scission of the rubber could not be ignored in the aging process. The relative content of the characteristic groups in rubber aging process was determined by ATR-FTIR. The thermal oxygenation degree of NBR could be determined by observing the relative changes in the content of characteristic groups at different aging times. The ATR-FTIR spectra of KB-1, KB-2, and NBR/NG-3 at different aging times are shown in Figure 15. The vibration absorption peak of –CH was at 2800–3000 cm^−1^, while 2235 cm^−1^, 1735 cm^−1^, and 1641 cm^–1^ corresponded to the absorption peaks of –CN, C=O, and C=C, respectively [22]. The relative change of each characteristic peak was small. Therefore, the internal standard method was used to semi-quantitatively calculate the integrated area of each characteristic peak, which indirectly characterizes the changes of each group at different aging times. The –CN group was selected as the internal standard characteristic group, because its content was almost unchanged during the aging process. Besides, the integrated peak area ratio of other groups to the area of the –CN peak was used as its relative intensity. The relative contents of the –CH, C=C, and C=O groups of the three samples at different aging times are shown in Figure 16, Figure 17 and Figure 18. The content of –CH gradually decreased, and the content of C=C gradually increased with the aging time [5], because the rubber chains gradually broke at the level of –CH, forming C=C during the aging process. With the aging time prolonged, the content of –CH and C=C in NBR/NG-3 changed more slowly than in KB-1 and KB-2. This indicated that NBR/NG-3 had a lower degree of chain scission during the aging process. It is worth noting that in the initial stage of the aging process (0–24 h), the changes in –CH, C=C, and C=O in KB-2 and NBR/NG-3 were small, because the commercial antioxidants of KB-2 and the anti-aging groups of NG had chemical anti-aging effects. After 24 h of aging, the contents of –CH, C=C, and C=O in KB-2 changed significantly faster than in NBR/NG-3, because the antioxidant of KB-2 gradually volatilized with aging. The anti-aging group grafted on the surface of NG could exert a chemical anti-aging function for a longer time. In addition, the lamellar NG was uniformly dispersed in the NBR, which could effectively prevent the diffusion of oxygen in the matrix.

## 4. Conclusions

Nitrogen-doped graphene oxide fillers were prepared using graphene oxide grafted by o-phenylenediamine. NG was evenly dispersed in the rubber matrix, as shown from the results of SEM, and could effectively inhibit the entry and diffusion of oxygen, achieving physical anti-aging. ATR-FTIR and TGA-DSC showed that NG fillers could significantly improve the aging resistance of NBR composites by inhibiting the cross-linking and breaking of the rubber chains. The interaction between NG and the rubber was strong, so that the phenolic zine and aromatic amine groups with anti-aging function were locked in the rubber matrix, achieving the goal of green chemical anti-aging. The aging resistance of NBR composites was obviously improved by synergizing the effect of the lamellar barrier of the NG filler and the chemical anti-aging effects of phenazine and aromatic amine groups.

## Figures and Tables

**Table 1:** Basic formulation of nitrile–butadiene rubber (NBR) composites.

Materials/phr ^a^	KB-1	KB-2	NBR-NG-1	NBR-NG-2	NBR-NG-3	NBR-NG-4	NBR-RGO
NBR	100	100	100	100	100	100	100
ZnO	5	5	5	5	5	5	5
Stearic acid	1	1	1	1	1	1	1
Sulfur	1.5	1.5	1.5	1.5	1.5	1.5	1.5
DM	0.5	0.5	0.5	0.5	0.5	0.5	0.5
Antioxidant D	0	1	0	0	0	0	0
NG	0	0	1	2	3	4	0
RGO	0	0	0	0	0	0	3

^a^ The digits below each composite name represent the parts of the filler per hundred parts (phr) of NBR rubber by weight. KB: pure NBR, NG: nitrogen-doped graphene oxide, RGO: reduction grapheen oxide, DM: dibenzothiazole disulfide, antioxidant D: N-Phenyl-2-naphthylamine.

**Table 2:** Definitions of the terms in Equations (1) and (2).

Character	Implication
V_r_	Crosslinking density of NBR composites
V_2_	Volume fraction of rubber phase in swelling NBR
χ	Interaction parameters between rubber and toluene
V	Molar volume of the solvent
m_1_	Mass of the NBR composite sample before swelling
m_2_	Mass of the NBR composite sample after swelling
m_3_	Mass of the rubber phase
ρ	Density of NBR
ρ_s_	Density of toluene

**Table 3:** Mechanical properties of NBR composites.

Sample	KB-1	KB-2	NBR				
			NG-1	NG-2	NG-3	NG-4	RGO-3
Stress at break (MPa)	1.82 ± 0.23	2.19 ± 0.17	3.11 ± 0.32	3.24 ± 0.25	4.20 ± 0.18	4.02 ± 0.27	3.02 ± 0.38
Elongation at break (%)	663 ± 34	648 ± 31	651 ± 25	640 ± 19	620 ± 29	624 ± 36	554 ± 43
